# Effects of Hemispheric Stroke Localization on the Reorganization of Arm Movements within Different Mechanical Environments

**DOI:** 10.3390/life11050383

**Published:** 2021-04-23

**Authors:** Laura Pellegrino, Martina Coscia, Camilla Pierella, Psiche Giannoni, Amel Cherif, Maddalena Mugnosso, Lucio Marinelli, Maura Casadio

**Affiliations:** 1 Department Informatics, Bioengineering, Robotics and Systems Engineering (DIBRIS), University of Genoa, 16126 Genoa, Italy; laura.pellegrino@edu.unige.it (L.P.); psiche.giannoni@gmail.com (P.G.); amel.cherif@iit.it (A.C.); maddalena.mugnosso@iit.it (M.M.); 2 Bertarelli Foundation Chair in Translational Neuroengineering, Ecole Polytechnique Federale de Lausanne, 1015 Lausanne, Switzerland; martina.coscia@virgilio.it; 3 Wyss Center for Bio- and Neuroengineering, 1202 Geneva, Switzerland; 4 Department of Neurosciences, Rehabilitation, Ophthalmology, Genetics, and Maternal and Children’s Sciences (DINOGMI), University of Genova, 16126 Genoa, Italy; lucio.marinelli@unige.it; 5 Robotics, Brain and Cognitive Sciences Department, Istituto Italiano di Tecnologia, 16163 Genoa, Italy; 6 Department of Neuroscience, Division of Clinical Neurophysiology, IRCCS Ospedale Policlinico San Martino, 16132 Genoa, Italy

**Keywords:** stroke subjects, upper limb, robot-based assessment, muscle synergies, spinal maps

## Abstract

This study investigated how stroke’s hemispheric localization affects motor performance, spinal maps and muscle synergies while performing planar reaching with and without assistive or resistive forces. A lesion of the right hemisphere affected performance, reducing average speed and smoothness and augmenting lateral deviation in both arms. Instead, a lesion of the left hemisphere affected the aiming error, impairing the feedforward control of the ipsilesional arm. The structure of the muscle synergies had alterations dependent on the lesion side in both arms. The applied force fields reduced the differences in performance and in muscle activations between arms and among populations. These results support the hypotheses of hemispheric specialization in movement control and identify potential significant biomarkers for the design of more effective and personalized rehabilitation protocols.

## 1. Introduction

Stroke is a prevalent cause of motor impairments worldwide and its incidence continues to rise [1,2,3]. To regain the ability to perform tasks that once were straightforward, after stroke, people undergo a reorganization of their neural control, involving their surviving networks [4]. Although different factors [5], such as the size and location of the lesion, can influence the reorganization patterns, to quickly regain independence, survivors often develop compensatory strategies that tend to be stereotypical [6,7,8] and energetically inefficient [9].

Upper limb impairments in the contralesional side of the body persist in 55–75% of chronic survivors [10,11]. The contralesional arm is indeed often characterized by motor deficits, including: (i) loss of fine motor control and deficits in motor planning and sensorimotor integration [12]; (ii) kinematics and dynamics trajectory abnormalities [12]; (iii) abnormal joint torque patterns [13,14] and joints temporal coordination [15,16,17], especially between elbow flexion and shoulder horizontal adduction [16]. This is associated with altered abnormal muscle activations and coordination patterns [18,19], such as increased activations [20] and prolonged delay in initiation and termination of the contractions [21]. Stroke survivors compensate for these deficits also with extensive use of the side of the body ipsilateral to the lesion [22].

To define motor and muscular alterations after stroke, many studies compared the contralesional arm of stroke survivors with the correspondent arm of controls [16,19,23] or with the ipsilesional arm of the same subject [14,20,24,25], considering the latter as ‘not impaired’. Although the arm ipsilateral to the lesion does not have evident impairments, it presents abnormalities [26,27,28,29], including altered control of distal movements [12,30,31], probably due to inappropriate temporal sequencing of muscle activity [12,32]. These changes could depend on the hemisphere directly affected by the lesion [12,29,33,34,35,36], since, in unimpaired subjects, the two hemispheres also play different roles in the motor control of trunk and arms [36,37,38,39]. Indeed, a lesion in the left hemisphere might determine coordination and motor planning deficits [38,39,40,41], while a lesion in the right hemisphere usually allows the generation of well-coordinated movements but followed by higher final position errors [38,39,40,41].

The purpose of this study is to systematically investigate how motor performance and muscle organization are altered by the hemispheric localization of the stroke lesion not only in the contralesional but also in the ipsilesional arm of chronic stroke subjects.

We used a planar robotic manipulandum for a quantitative and repeatable assessment of upper limb motion strategies and to provide three different mechanical environments to increase the variability of the reaching task. Therefore, we compared the ipsilesional and contralesional side of stroke survivors with right (RBD) and left (LBD) brain damage with the corresponding body side of aged-matched right-handed unimpaired controls. We focused on two methodologies enabling the analysis of the activity from multiple upper limb muscles: spinal maps and muscle synergies.

The spatiotemporal activity of motoneuronal (MN) pools belonging to the spinal cord, named a “spinal map”, represents the spatiotemporal organization of the electromyographic (EMG) signals at the level of the spine and it is a useful tool to explore overall muscle organization. It has been adopted to investigate muscle and spinal activity in lower and upper limb motor tasks in unimpaired subjects [42,43,44,45,46,47,48] and in neuropathologies, such as spinal cord injury (SCI) [49,50] and stroke [51,52]. Despite their sensitivity to pathological features, so far, the spinal maps have not been explored in depth for upper limbs of stroke survivors.

The term “muscle synergy”, instead, has been used to define a set of co-activated muscles [53], and, in stroke, it has gained clinical relevance because their loss of integrity is considered a physiological marker of motor cortical damage. Specifically, their organization on the contralesional side depends on the level of impairment and on the onset of the cerebrovascular accident [18,54,55,56,57]. However, muscle synergy changes due to stroke events in specific hemispheres have not been extensively explored [18].

Our results suggest that, compared to controls, in the stroke population, upper limb motor performance, spinal maps and muscle synergies in the ipsilesional side are modified, although less altered than in the contralesional side. The RBD and LBD subjects present differences in motor performance and in the organization of muscle synergies, supporting the hypotheses of hemispheric specialization in movement control. The presence of force fields reduced differences in performance and in muscle activations, both between the two arms and among different populations.

## 2. Materials and Methods

### 2.1. Subjects

Twenty-five chronic stroke subjects with a single cerebrovascular accident and consequent hemiparesis were enrolled in accordance with the following requirements: chronic stroke (>6 months after stroke event), Modified Ashworth Scale Bohannon & Smith (MAS) related to shoulder and elbow ≤ 3 [58], no botulinum toxin injection in the paretic arm in the last 3 months and no evidence of severe cognitive or language dysfunctions that would have interfered with the ability to understand instructions, i.e., mini-mental state examination ≥ 25 [59]. Demographic and clinical data for each stroke subject are listed in Table 1. Each stroke survivor was evaluated using the Fugl-Meyer scale for upper extremity (FMA-UE, maximum score of 66) [60]. The stroke population showed heterogeneous impairments, with the FMA-UE score ranging from 5 to 58.

Twenty-five unimpaired control subjects (C) were recruited to serve as controls matched in terms of their sex and age with the stroke subjects. In particular, each stroke survivor had a corresponding control subject. The controls and stroke subjects were similar in terms of age (z = −0.1831, *p* = 0.681) and sex (χ^2^(2) = 0.000, *p* = 1.000). All control subjects were right-handed according to the Edinburgh Medical Research Council handedness scale [61] and all survivors reported to be right-handed before the occurrence of the stroke event. All subjects were naïve to the proposed tasks and had no problems of visual integrity, i.e., they could clearly see the information—target and cursor positions—that was displayed on the computer screen.

Among all stroke subjects, 14 subjects had a left hemiparesis (RBD) and 11 right hemiparesis (LBD). Both stroke groups (LBD and RBD) were sex- and age-matched (RBD: 9M–5F, age 61 ± 9 years and LBD: 7M–4F, age 60 ± 11 years, age: z = −0.1921, *p* = 0.8470, sex: χ^2^(2) = 0.001, *p* = 0.973) and had similar impairment as assessed by the FMA-UE scale (score (max 66): 27.4 ± 17.9 and 26.7 ± 17.8, respectively; z = −0.1917, *p* = 0.8480) and by the MAS scale at the elbow and shoulder (MAS shoulder RBD: 2 ± 0.62, LBD: 1.091 ± 0.801 z = −2.5498, *p* = 0.018, MAS elbow RBD: 1.679 ± 0.890 LBD: 1.546 ± 1.193 z = −0.1397, *p* = 0.888). All stroke subjects reported to be right-handed before the occurrence of the stroke event. All subjects were naïve to the proposed tasks and had no problems of visual integrity, i.e., they could clearly see the information—target and cursor positions—that was displayed on the computer screen.

The experimental procedures were carried out at the Department of Informatics, Bioengineering, Robotics and Systems Engineering (DIBRIS) of the University of Gen-oa, and the stroke survivors were recruited among the outpatients of the Department of Neuroscience, Rehabilitation, Ophthalmology, Genetics, Maternal and Child Health (DINOGMI) of the University of Genoa.

The study was approved by the local Ethical Committee (Comitato Etico ASL3 Genovese, 09-04-2013, REGISTRO ASL 13/13) and conformed to the ethical standards of the 1964 Declaration of Helsinki. Each subject provided written informed consent to participate in the study and to publish individual data.

### 2.2. Experimental Set-Up

Subjects grasped the handle of a planar robotic manipulandum [62] characterized by low friction, low inertia, zero backslash, large elliptical workspace (80 cm × 40 cm) and actuated by a pair of direct-drive brushless electric motors. The robot encoders recorded the end-effector position, and the robot motors provided the interaction forces.

Subjects sat on a chair, with their arm and wrist restrained by means of suitable holders, and they grasped the handle of the planar robotic manipulandum. The position of the seat was also adjusted in such a way that, with the cursor pointing at the center of the workspace, the movements were restricted to the horizontal plane, with no influence of gravity. A chair provided a secure back support and two belts prevented appreciable trunk movements. A 19″ LCD computer screen was placed vertically in front of the subjects, around one meter away, at eye level. The scale factor of the display was 1:1, i.e., 1 cm on the screen corresponded to 1 cm in the robot workspace.

Stroke subjects and age- and sex-matched controls performed reaching movements with the left and right arm in different mechanical environment, including:

(i) *Assistive force field (AF*): a constant assistive force field attracted the hand of the subjects toward the peripheral target (force amplitude: 5N). The assistance was maintained constant because we wanted to provide an environment where subjects received a haptic cue of the target and assistance that did not vary depending on the hand position, as in [63].
(ii) *Null force (NF)*: the hand of the subjects moved unimpeded by external forces.
(iii) *Resistive force field (RF):* a resistive force field attracted the hand of the subjects toward the center of the workspace, i.e., an elastic force opposed the subjects’ movements toward the peripheral targets (linear spring stiffness coefficient was 15 N/m). In the center of the workspace, i.e., in the center of the home target, the force field was 0 N. With this force field, we wanted to simulate the pulling of a virtual spring.

**Figure 1:**
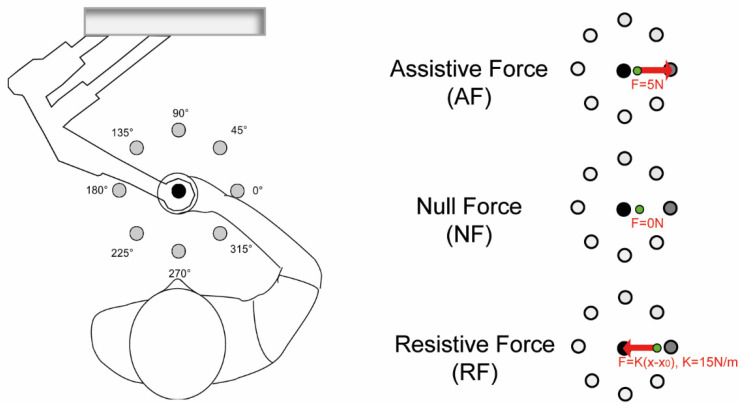
Left panel: The experimental setup. Subjects held the handle of the planar robotic manipulandum and made reaching movements towards targets presented on a computer screen placed in front of them. The targets are shown as grey circles. Right panel: Experimental tasks. Tasks were performed in three different mechanical environments: assistive force (AF), null force (NF) and resistive force (RF). In the latter environment, the force was 0N in the origin of the workspace (x0 = [00]), i.e., in the center of the home target. The black dot is the starting target; in dark grey, the target to reach is shown, and the green dot is the cursor corresponding to the hand position. The red arrows indicate the direction of the force exerted by the robot.

Subjects were asked to reach targets positioned in 8 directions (0°, 45°, 90°, 135°, 180°, 225°, 270°, 315°) equally spaced from a central target (distance: 14 cm) (Figure 1). The different directions and mechanical environments were adopted to add variability in the controlled motor task, to ensure good representation of reaching movements and to use representative task conditions usually adopted for robotic-aided rehabilitation. Targets were displayed as round green circles (1 cm radius) against a black background. Each target was presented five times (5 × 8 = 40 center-out movements per task) in random order. The current position of the hand was continuously displayed, as a yellow circle (0.5 cm radius), during the execution of the task in the three different mechanical environments.

Stroke subjects started the experiment with their contralesional arm. Control subjects who were matched with stroke subjects with an LBD lesion started the experiment with the right arm, while control subjects matched with stroke subjects with an RBD lesion started the experiment with the left arm. The tasks in the three different environments were presented in random order within each arm. Subjects were asked to reach the targets as accurately as possible, without time constraints; thus, they performed the task at their self-selected speed.

When comparing the two arms, we considered equivalent directions that corresponded in joint coordinates. In the endpoint space, the left-hand trajectories and the directions of action of muscle activities, spinal maps and muscle synergy activations were mirrored at the midline to be compared with the corresponding right-hand trajectories and muscle directions. Thus, the target directions indicated in the text and figures as 315°, 0°, 45° corresponded to rightward movements of the right-hand and leftward movements of the left-hand; vice versa, the target directions 135°, 180°, 225° corresponded to leftward movements of the right-hand and rightward movements of left-hand.

Muscle activity was recorded with surface electrodes for electromyography (CometaWavePlus wireless EMG system, Cometa Srl, Milano, Italy). Surface EMG signals were recorded from the following 16 upper limb muscles: Triceps Brachii long head (TB-long), Triceps Brachii lateral head (TB-lat), Biceps Brachii short head (BB-short), Biceps Brachii long head (BB-long), brachioradialis (BRAD), pronator teres (PRON), infraspinatus (INFR), latissimus dorsi (LAT), upper trapezius (TRAP), rhomboid major (RHOM), pectoralis major (PECT), anterior deltoid (DELT-ant), medial deltoid (DELT-mid), posterior deltoid (DELT-post), extensor carpi radialis (EXTE) and flexor carpi radialis (FLEX). Electrodes were placed according to guidelines of the Surface Electromyography for the Non-Invasive Assessment of Muscles European Community project—SENIAM [64]—and to anatomical guidelines [65]. EMG electrode placement was performed according to recommendations for minimizing cross-talk from adjacent muscles [64]. Additionally, the absence of cross-talk among muscles was tested through visual inspection of the EMG signals while performing suitable movements at the moment of electrode placement.

The protocol required a minimum of a two-minute break between each task. Subjects could rest whenever they asked for a break and for as long as they needed; the experimental sessions lasted around two hours. All subjects were able to complete the proposed tasks.

### 2.3. Data Analysis

#### 2.3.1. Behavioral Indicators

Movement trajectories were acquired at 60 Hz. The *x* and *y* components were smoothed with a 6th order Savitzky–Golay filter (cut-off frequency: ~11 Hz), which was also used to estimate the subsequent derivatives: velocity, acceleration and jerk. We only considered the movements going from the central to the outer targets.

The movement onset was defined as the first time instant when the cursor speed exceeded a threshold of 10% of maximum peak speed [66]. The movement ended when the cursor was inside the target and the speed underwent the same threshold.

We computed the following indicators:

- Average speed (m/s): average speed of the cursor movement.
- Smoothness index (dimensionless): the square root of the jerk (norm of the third time derivative of the trajectory), averaged over the overall movement duration, computed according to [67]. This indicator is dimensionless and allows comparison of movements with different duration and path length [68]. High values of this indicator correspond to jerky movements.
- Lateral deviation (m): the maximum lateral distance between the cursor trajectory and the nominal trajectory [69]. It indicates accuracy in movement execution.
- 100-ms aiming error (deg): the angular difference between the target direction and the actual movement direction, estimated in the first 100 ms of the movement [70]. It indicates the ability to plan movements, i.e., the performance of the feedforward component of control.

#### 2.3.2. EMG Processing

EMG signals were acquired at 2 kHz, band-pass-filtered (30–550 Hz), rectified and low-pass-filtered (cutoff: 10 Hz) to obtain the EMG envelopes [18]. To correct the inter-arm and inter-subject EMG amplitude differences due to electrode placement and skin impedance, the envelope of each muscle signal was normalized by its median value obtained during the center-out movements of all the tasks, i.e., for each subject, we computed the median over all his/her EMG signals considered in the study. This allowed us to compare activation profiles across directions and different environments. The normalization based on the median value instead of the maximum is more robust against high-amplitude spikes arising from noise [71]. The normalized EMG envelopes for each subject, arm, task and repetition were segmented according to the 8 directions. In order to compute the spinal maps, the normalized EMG envelope of each muscle related to each trial was resampled on 100 time points.

#### 2.3.3. Spatiotemporal Motoneuronal Activity

Pre-processed EMG signals were used to estimate the correspondent spatiotemporal organization of the MN activity in the spinal cord [42,52,72]. To characterize the spinal motor output, EMG activity was mapped onto the estimated location of MN pools innervating the different muscles of the upper limb as reported by Kendall [73]. The map was limited to levels between C2 and T1 in relation to the set of recorded muscles, as reported in Table 2. The metric adopted to describe the similarity between two spinal maps was the 2D Pearson’s correlation coefficient (ρ_2D-GROUP_) [42,48,51]. More specifically, the averaged value obtained by comparing each stroke survivor, each arm and task with the correspondent control subjects was considered representative of the degree of similarity between the LBD or RBD group and their respective control groups. To obtain a reference value for the degree of similarity, the Pearson’s correlation coefficient was computed for the EMG signals within the same arm and task for the control subjects (ρ_2D INTRA-GROUP_) [74,75]. In the same way, for each task, we estimated the similarity between arms (ρ_2D-ARM_) within groups for each population [74,75].

#### 2.3.4. Muscle Synergies

For each subject, task and arm, we applied the non-negative matrix factorization (NNMF) algorithm [71,76,77,78,79] to a matrix obtained by concatenating for each muscle (rows) the normalized EMG envelopes related to the eight directions (columns) averaged over the five repetitions. The NNMF algorithm extracts from the EMG envelopes a defined number of positive components or muscle synergies, represented by a matrix of weights (W) accounting for the participation of each muscle in each synergy, and a matrix of activation coefficients (H) representing the timing of activity of each muscle synergy.

For each subject, to objectively determine the minimum number of muscle synergies required to reconstruct each data set, we used the common or the higher number obtained from two different methods based on the inspection of the R^2^ curve that represents the fraction of total variation explained by the synergy model [78]. The first method estimated the minimum number of synergies that achieved a R^2^ > 90% [78,80]. The second method was based on the detection of a change in the slope of the R^2^ curve [80]. For the second method, a series of linear regressions were performed on the portions of the curve included between the N-synergy (N = 1 to 16) and its last point (i.e., 16th synergy). N was then selected as the minimum value for which the mean squared error of the linear regression was less than 10^−4^. In case of mismatch between the two criteria, the larger N was chosen [80]. In order to simplify the analysis, the same number of muscle synergies was retained across subjects within the same arm, task and group to compare the weights and activation coefficient vectors of muscle synergies among populations; the number was established as the rounded average across subjects [79].

To compare muscle synergies among tasks, arms and groups, the weight coefficients of each muscle synergy were ordered according to their matching with a set of reference weight coefficients [42,74] by using the highest normalized scalar product between the two vectors for each task [81]. The following steps describe how we obtained the reference muscle synergies, for each condition and each arm separately. Since we observed that the number of muscle synergies was equal between arms and groups, we created a set of reference muscle synergies for each task, first by pooling together the weight coefficients related to right and left arm for control subjects. Then, according to Cheung et al. [18], we used a hierarchical clustering procedure based on the minimization of the Minkowski distance between vectors to categorize them. The number of clusters was equal to the number of muscle synergies extracted for each task. We obtained the set of reference muscle synergies by averaging the synergy vectors within each cluster. Then, we ordered the synergy vectors from each subject, in each task and in each arm separately, with respect to the set of reference muscle synergies.

To assess the similarity between groups in all tasks and arms for the weight coefficients, we computed the scalar product (DOT_GROUP_) between the synergy vectors of each arm and task, and then we calculated the mean values across subjects and synergies. In the same way, we estimated the similarity between sides (DOT_ARM_) within each group for the AF, NF and RF task. We evaluated the similarity of the activation coefficients of muscle synergies by using the Pearson correlation [23,82]. Analogously to the weight coefficients, we estimated the similarity between groups (r_GROUP_) and between arms (r_ARM_) in all tasks. To obtain a reference value to assess the degree of similarity between groups, the weight and the activation coefficients of each control subject were compared, respectively, with the weight and activation coefficients of all other controls and then averaged across muscle synergies and across individuals (DOT_INTRA-GROUP_ and r_INTRA-GROUP_, respectively) [74,75].

#### 2.3.5. Statistical Analysis

To verify that control and stroke groups (LBD and RBD) were not different in terms of age and that the LBD and RBD group were similar in terms of motor impairment, we used the Wilcoxon test for between-group comparisons for both the clinical scales and age analysis. Additionally, we used the chi-squared test for verifying that groups were not different in terms of sex.

To test the hypothesis that the behavioral performance (average speed, smoothness index, lateral deviation, aiming error) and the muscle activation and synergies (ρ_2D-GROUP_, number of muscle synergies, DOT_GROUP_, r_GROUP_) for the ipsilesional and for the contralesional arm differed between stroke subjects and the corresponding arm of control subjects in each task, we ran a repeated-measures ANOVA with one within-subjects factor, “task” (AF, NF and RF), and one between-groups factor, “pathology” (stroke subjects and controls).

To test the hypothesis that LBD and RBD have different behavioral performance and muscle activation and synergies in both arms and in different tasks, we ran a repeated-measures ANOVA with two within-subjects factors, “arm” (contralesional and ipsilesional arm) and “task” (AF, NF and RF), and one between-groups factor, “side of lesion” (LBD and RBD).

Furthermore, to investigate the similarity between the two arms of each subject in terms of spinal maps (ρ_2D-ARM_) and muscle synergies (DOT_ARM_, r_ARM_), we ran a repeated-measures ANOVA with one within-subjects factor, “task” (AF, NF and RF), and one between groups factor, “pathology” (C, LBD, RBD).

We defined as outliers the datapoints that followed outside the region of ±2 standard deviation of the mean. For each subject, when an outlier was identified for one variable, the correspondent trial was removed from both EMG and kinematic analysis.

Prior to statistical testing on each variable, we tested the hypothesis that all the indicators were normality distributed with the Kolmogorov–Smirnov test. We used Mauchly’s tests to verify the sphericity assumption.

The hypothesis of normality was verified for all indicators (*p* > 0.05). Instead, in all the cases where the sphericity assumption was not verified, we adopted the Greenhouse–Geisser correction (see Supplementary Material). Post-hoc analysis (Fisher’s LSD test) with Bonferroni correction for multiple comparisons was used to verify statistically significant differences obtained with repeated-measures ANOVA. The significance level was set at *p* < 0.05. The statistical analysis was performed within the Statsoft environment (Statistica software 7.1).

In all the figures of the Results section, we report the significance obtained with statistical analysis for the comparison i) between stroke survivors and controls, for each arm (i.e., the significance of the main effect for the ‘pathology factor’, at the top of each figure panel); ii) between LBD and RBD groups (separately for each side of the body and for each environment condition (post-hoc analysis, inside each figure, at top of each pair of columns)). In all cases, asterisks represent *p* < 0.05.

## 3. Results

### 3.1. Stroke Subjects Had Different Performance than Controls and the Differences Depended on the Side of the Brain Lesion: A Lesion on the Right Hemisphere Determined Worse Performance in both Arms in Terms of Average Speed, Smoothness and Lateral Deviation of the Overall Trajectory. Instead, a Lesion on the Left Hemisphere Affected the Feedforward Control of the Movements for the Ipsilesional Hand, as Highlighted by the Aiming Error

**Figure 2:**
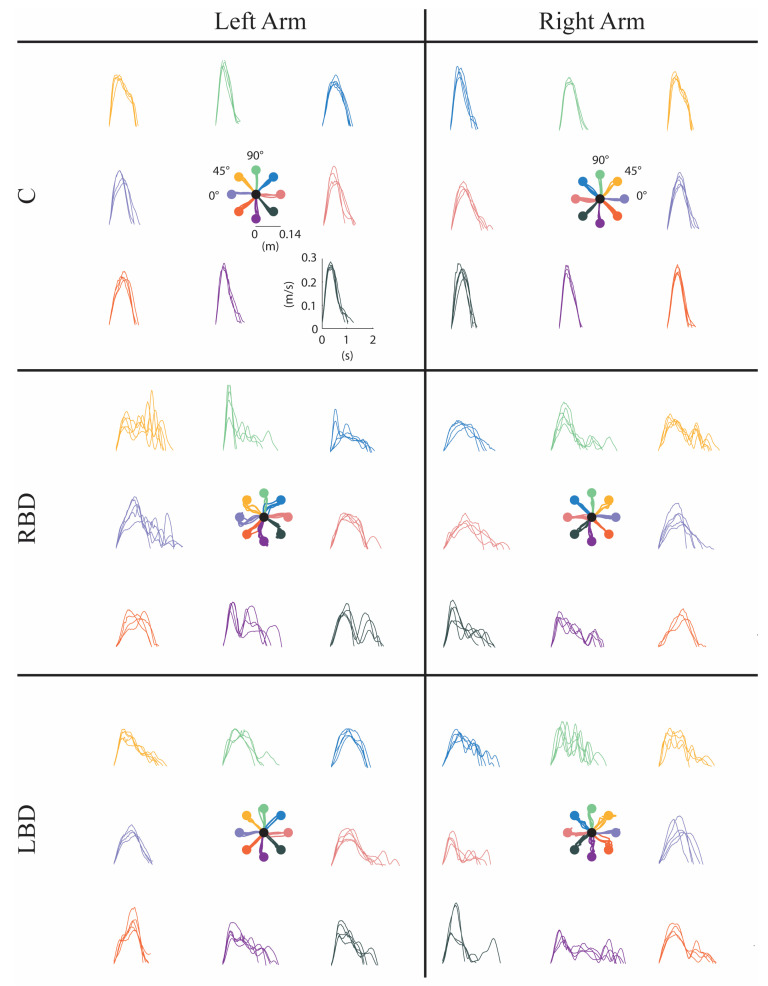
Movement trajectories (at the center of each panel) and speed profiles in the null force task (NF) for a control (C, first row), a right brain damage (RBD, second row) and a left brain damage (LBD, third row) subject. Data from the left and the right arm are reported in the left and right panels, respectively. The left arm of the RBD subject and the right arm of the LBD subject correspond to the contralesional (paretic) arm. The right arm of the RBD subject and the left arm of the LBD subject correspond to the ipsilesional (non-paretic) arm. The correspondence between movement trajectories and speed profile for each direction is color-coded. Directions displayed with the same color correspond to the same movements in joint space.

Stroke subjects had worse performance than controls. All stroke subjects had cursor trajectories characterized by multiple large corrective movements in the second part of the trajectories in both arms, as shown in Figure 2 for a representative stroke subject.

**Figure 3:**
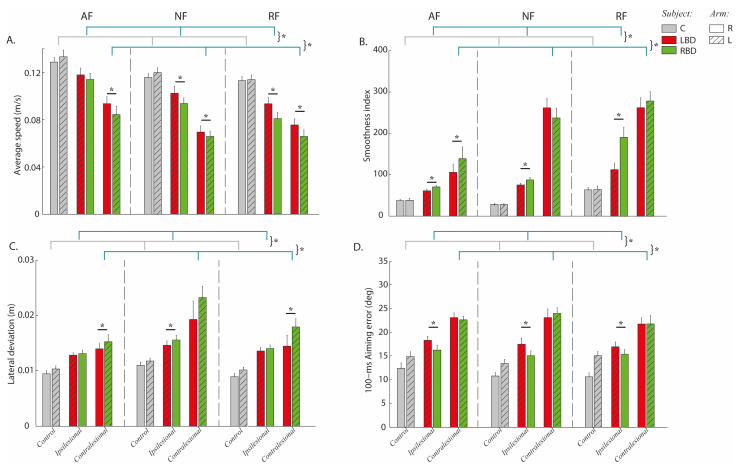
Stroke’s hemispheric localization affects motor performance: a lesion of the right hemisphere affected performance, re-ducing average speed and smoothness and augmenting lateral deviation in both arms. Instead, a lesion of the left hemi-sphere affected the aiming error, impairing the feedforward control of the ipsilesional arm. Behavioral indicators: aver-age speed (**A**), smoothness index (**B**), lateral deviation (**C**) and 100-ms aiming error (**D**) computed from the cursor tra-jectories. Controls (C, grey bars) and subjects with Right Brain Damage (RBD, green bars) and Left Brain Damage (LBD, red bars) moved in presence of assistive (AF), resistive (RF) and in absence of external force (NF). The movements were performed with the right (bars with uniform color) and left (bars with diagonal lines) arm. Error bars indicate the stand-ard error of the indicators. The contralesional arm (paretic) corresponds to the left (L) arm in RBD subjects and to the right (R) arm in LBD subjects. The ipsilesional arm (non-paretic) corresponds to the right (R) arm in RBD subjects and the left (L) arm in LBD subjects. * indicates significant differences (*p* < 0.05).

As expected, stroke subjects differed from controls, being slower (F(2,47) = 40.23, *p* < 0.001), less smooth (F(2,47) = 14.24, *p* < 0.001) and less accurate both for the 100-ms aiming error (F(2.47) = 20.47, *p* = 0.021) and for the maximum lateral deviation of the trajectory (F(2,47) = 13.27, *p* = 0.001) in the contralesional arm. Moreover, in the ipsilesional arm, their movements were slower (F(2,47) = 33.46, *p* < 0.001), less smooth (F(2,47) = 13.86, *p* < 0.001) and less accurate both in terms of 100-ms aiming error (F(2.47) = 11.28, *p* = 0.032) and lateral deviation (F(1,47) = 39.32, *p* = 0.001) than in the correspondent controls’ arm; see Figure 3A–D. The difference between the motor performance of stroke subjects and controls was task-dependent for both the contralesional arm (interaction effect: pathology x task; average speed F(4,94) = 13.53, *p* < 0.001, smoothness index F(4,94) = 17.41, *p* = 0.02, 100-ms aiming error F(4,94) = 15.51, *p* = 0.03 and lateral deviation F(4,94) = 16.04, *p* = 0.004) and the ipsilesional arm (interaction effect: pathology x task; average speed F(4,94) = 10.25, *p* = 0.002, smoothness index F(4,94) = 12.72, *p* = 0.001, 100-ms aiming error F(4,94) = 15.64, *p* = 0.022 and lateral deviation F(4,94) = 12.27, *p* = 0.011). Specifically, this difference was higher when moving in the absence rather than in the presence of forces exerted by the robot.

Moreover, LBD and RBD subjects had different performance (side of lesion effect: average speed F(1,23) = 7.45, *p* = 0.012, smoothness index F(1,23) = 6.12, *p* = 0.001, 100-ms aiming error F(1,2310) = 6.42, *p* = 0.031 and lateral deviation F(1,23) = 9.32, *p* = 0.023; see Figure 3A–D) and also this difference was task-dependent (interaction effect: site of lesion x task: *p* < 0.001 for all indicators). Indeed, the RBD group in most tasks was slower (post-hoc NF: *p* = 0.003, AF: *p* = 0.002 and RF: *p* = 0.001) and less smooth (post-hoc NF: *p* = 0.172 AF: *p* = 0.011 and RF: *p* = 0.031) with the contralesional arm. Similarly, the RBD group had also lower average speed (post-hoc NF: *p* = 0.003, AF: *p* = 0.100 and RF: *p* = 0.001) and higher smoothness index (post-hoc NF: *p* = 0.004, AF: *p* = 0.021 and RF: *p* < 0.001) for the ipsilesional arm.

As for the accuracy during movement execution, the RBD group tended to have higher lateral deviation than the LBD group with the ipsilesional arm; this difference was significant only in the null force condition (post-hoc: *p* = 0.034). On the contrary, the contralesional arm of the RBD group had significantly higher lateral deviation than the LBD group in the presence of assistive (post-hoc *p* = 0.020) and resistive force (post-hoc *p* = 0.004). When looking at the feedforward component of the control, the aiming error of the LBD group was found to be less accurate than RBD (*p* < 0.03 for all tasks) for the ipsilesional arm in all tasks.

### 3.2. Stroke Subjects Had Altered Spinal Maps in both Arms: Their Motoneuronal Activity Was Prolonged in Time Independently on the Side of the Brain Lesion. In the Contralesional Arm, the Activations Were Extended Rostral Towards C2, C3 and C4 Segments. The Presence of a Resistive Force Field Increased the Similarity of Spinal Maps, between Arms and among Subject Populations

**Figure 4:**
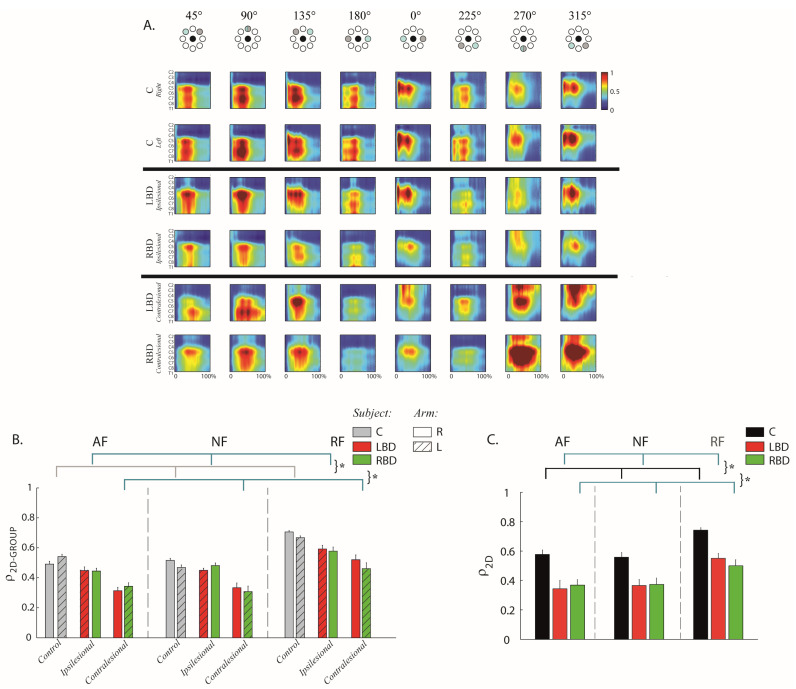
Stroke subjects had altered spinal maps in both arms. However, the presence of a resistive force field increased the similarity of spinal maps, between arms and among subject populations. (**A**) The spinal maps obtained for the eight targets in the resistive force (RF) task. The first two rows refer to the right and left arm of controls (C), the third and the fourth rows to the ipsilesional arm (non-paretic) of the left brain (LBD) and right brain (RBD) damage subjects, while the fifth and sixth rows refer to their corresponding contralesional (paretic) arms. On the *x*-axis, the movement duration is represented as a percentage. Spinal maps refer to equal movements in the joint space, i.e., for each column, the top panel indicates the corresponding target directions (grey target) for the right arm, while the corresponding target directions of the left arm were mirror symmetric with respect to the vertical midline. (**B**) Mean and standard error of the inter-group similarity (ρ_2D-GROUP_) between control (C) and stroke subjects (LBD, red bars; RBD, green bars) in presence of assistive (AF), resistive (RF) and in absence of external force (NF) for the right (bars with uniform color) and left (bars with diagonal lines) arm. The bars with uniform color (right arm) and with diagonal lines (left arm) reflect the intra-group degree of similarity in the control group. The contralesional arm (paretic) corresponds to the left (L) arm in RBD subjects and to the right (R) arm in LBD subjects. The ipsilesional arm (non-paretic) corresponds to the right (R) arm in RBD subjects and the left (L) arm in LBD subjects. (**C**) Mean and standard error of the between arm similarity (ρ_2D-ARM_) for controls (C, black bar), left (LBD, red bar) and right (RBD, green bar) brain damaged in presence of assistive (AF), resistive (RF) and in absence of external force (NF). * indicates significant differences (*p* < 0.05).

In controls, the main spatiotemporal motoneuronal activity related to upper limb planar reaching movements was observable between 10% and 60% of the movement duration in all tasks. This activation was localized caudally (between C5 and T1) for the distal/contralateral directions of the hand movements (i.e., 45°, 90°, 135° and 180°) and rostrally (between C4 and C7) for the proximal/ipsilateral directions (i.e., 225°, 270°, 320° and 0°) for all conditions; see Figure 4A.

Stroke subjects had a different organization of the spinal maps with respect to controls; these observations were confirmed by the statistical analysis using the 2D Pearson’s correlation coefficient between two different spinal maps (ρ_2D-GROUP_-F(2,47) = 65.48, *p* < 0.001 for the contralesional and F(2,47) = 58.66, *p* < 0.001 for the ipsilesional arm; see Figure 4B). In particular, the contralesional arm of the stroke subjects had a remarkable MN activity prolonged in time, i.e., from 10% to around 90% of the movement duration for movements in the proximal and proximal–ipsilateral directions (i.e., 270° and 315°, see Figure 4A). This activation was extended rostrally towards the C2, C3 and C4 segments, normally not so involved during planar movements in the absence of gravity (Figure 4A). These spinal segments (C2, C3 and C4) innervate mainly the TRAP, BB-long and BB-short (see Table 2). The prolonged spinal activity in the contralesional arm was also caudal (between C7–C9) for the distal–contralateral directions (i.e., 90° and 135°, see Figure 4A).

In the ipsilesional arm, the abnormal prolongation of spinal map activity was less marked than in the contralesional arm, but still detectable (Figure 4B) mainly in C5 and C6 for the distal movement direction (90°, Figure 4A), in all segments for the proximal and proximal contralateral direction (225° and 270°, Figure 4A) and in C4 to C8 in the proximal ipsilateral direction (315°, Figure 4A).

Spinal maps of LBD and RBD subjects were similar for both arms (side of lesion effect: F(1,23) = 2.30, *p* = 0.498; side of lesion x arm effect: F(1,23) = 3.52, *p* = 0.874; Figure 4B). The comparison of the spinal maps between the two arms of each subject (ρ_2D-ARM_) confirmed all these findings. The MN activity of the two arms was more similar for the controls than for the stroke subjects (pathology effect: F(2,47) = 11.23, *p* = 0.001) and this similarity was equivalent for RBD and LBD subjects (post-hoc LBD vs. RBD: *p* = 0.654); see Figure 4C.

Finally, the mechanical environment determined significant differences in the spinal maps both when comparing controls and stroke subjects (ρ_2D-GROUP_-ipsilesional arm task effect: F(2,47) = 10.45, *p* = 0.010; contralesional arm task effect: F(2,47) = 11.24, *p* = 0.014), and when comparing the two arms of the same subject (ρ_2D-ARM_-task effect: F(2,47) = 21.65, *p* = 0.014; post-hoc AF vs. RF and NF vs. RF: *p* < 0.001): in both cases, the similarity of the spinal maps was always higher in the presence of the resistive force field than in the AF or NF environments; see Figure 4B,C.

### 3.3. Muscle Synergies of Chronic Stroke Subjects Differed from Those of Controls in both Arms. In Absence of Force Fields, the RBD Group Had Weight Coefficients of Muscle Synergies Less Altered than the LBD Group in both Arms and More Similar between the Two Sides of the Body. The Presence of a Resistive Force Field Increased the Similarity of the Synergy Activations, between Arms and among Subject Populations

There was not a difference in the number of synergies between controls and stroke subjects in both the ipsilesional (F(2,47) = 2.4, *p* = 0.478) and contralesional arm (F(2,47) = 3.41, *p* = 0.640), between sides (side of lesion: F(1,23) = 2.24, *p* = 0.41) and among tasks (task effect: in ipsilesional arm F(2,94) = 1.91, *p* = 0.254 and contralesional arm F(2,94) = 1.91, *p* = 0.254); see Supplementary Material Figure S2. Therefore, according to the selected threshold criterion to define the number of synergies, and in order to simplify the comparison of muscle synergies for the different cases, we extracted five muscle synergies for each group (i.e., C, LBD and RBD), arm and task.

#### 3.3.1. Organization of Muscle Synergies: Weight Coefficients

**Figure 5:**
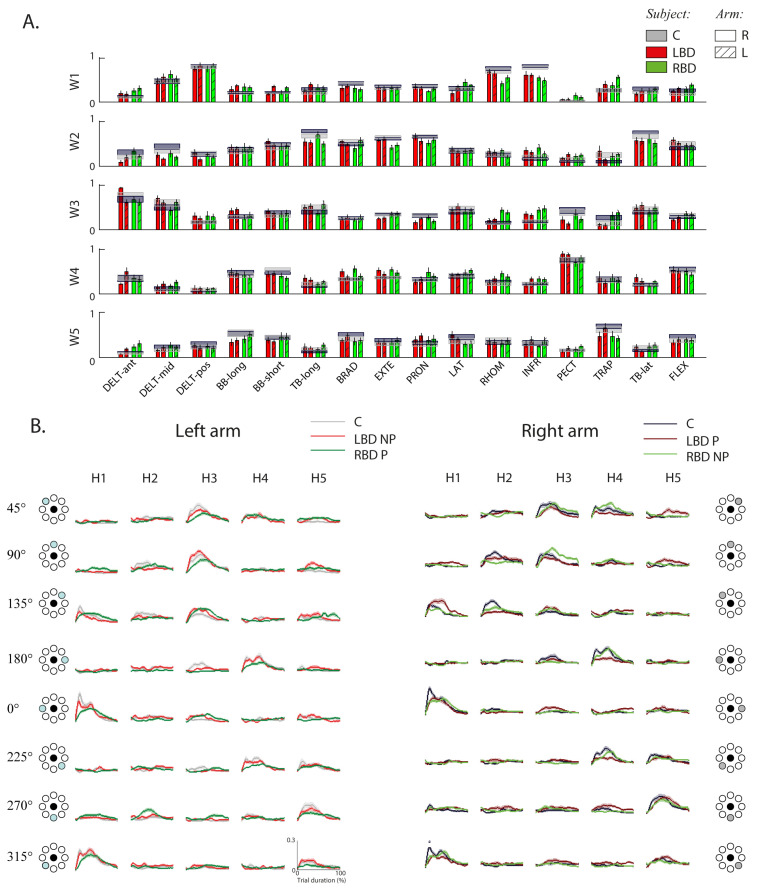
Stroke’s hemispheric localization affects the structure (weights) and the activation profiles of the muscle synergies in both arms. Weight and activation coefficients of muscle synergies during the resistive force task (RF). (**A**) Weight coefficients (W) for all muscle synergies (W1 to W5) for the two arms (right: bars with uniform color; left: bars with diagonal lines). The weights and the activation profiles of the muscle synergies result from the decomposition of muscle signals, each normalized by its median value. Weights represent the relative weighting (from 0 to 1) of each muscle within each synergy, where 0 is no contribution to the synergy and higher values indicate higher contribution. Controls (C) and stroke subjects with right brain damage (RBD) and left brain damage (LBD) are shown with different colors, as indicated in the legend. The error bars represent the standard error. (**B**) Mean activation profiles (H) for C (grey), RBD (green) and LBD (red) for all muscle synergies (H1 to H5) in the right (right panel) and left (left panel) arm. The shaded area indicates the standard error. Right and left activation profile coefficients refer to equal movements in the joint space, i.e., for each column, the left panel indicates the corresponding target directions (grey target) for the right arm, while the corresponding target directions of the left arm were mirror symmetric with respect to the vertical midline. * indicates significant differences (*p* < 0.05).

In both populations, the organization of muscle synergies was the following (Figure 5A,B):

- Synergy S1 principally involved the DELT-pos, DELT-mid, RHOM and INFR;
- Synergy S2 principally involved the TB-lat, TB-long, BRAD, PRON and EXTE;
- Synergy S3 principally involved the DELT-ant and DELT-mid;
- Synergy S4 principally involved the PECT, FLEX, BB-long and BB-short;
- Synergy S5 was a muscle-specific synergy dominated by the activity of the TRAP, with a minor contribution from the other muscles.

**Figure 6:**
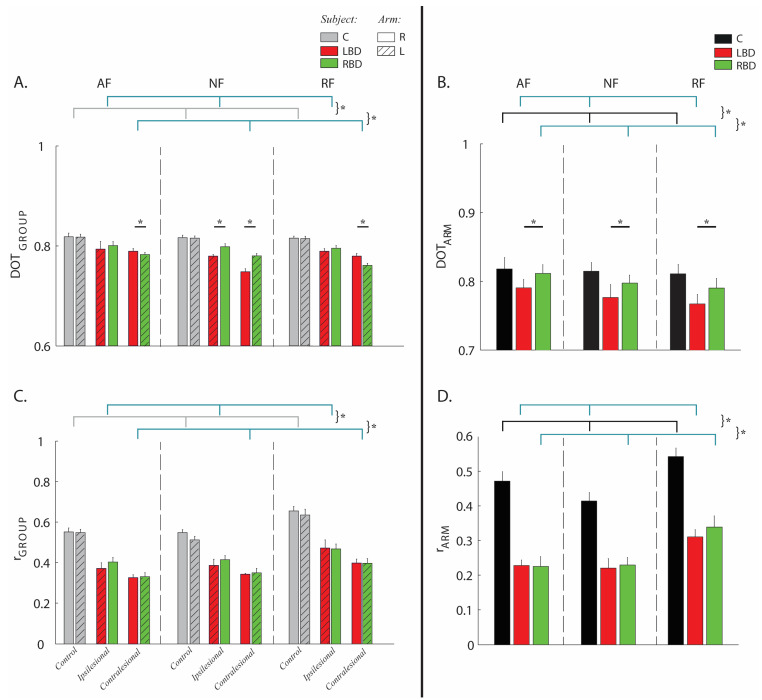
Comparison of weight and activation coefficients of the muscle synergies. RBD group had weight coefficients less altered than the LBD group in both arms and more similar between the two sides of the body. The force fields reduced the differences in muscle activities between arms and among populations. Panels (**A**,**B**): comparison of weight coefficients by the scalar product (DOT). Panels (**C**,**D**): comparison of activation coefficients by Pearson correlation (r). Left column: stroke subjects (LBD, red bars; RBD, green bars) compared to matched controls (C, grey bars), i.e., inter-groups indicator for the right (bars with uniform color) and left (bars with diagonal lines) arm in presence of assistive (AF), resistive (RF) and in absence of external force (NF) compared to the same indicator computed intra-group (grey bars) in the controls. Right column: comparison between the two arms of the same subject (C, black bar), left (LBD, red bar) and right (RBD, green bar) brain damaged in presence of assistive (AF), resistive (RF) and in absence of external force (NF). The error bars indicate the standard error. * indicates significant differences (*p* < 0.05).

The weight coefficients (W) described the contribution of each muscle to the synergies, i.e., they represent the relative weighting (from 0 to 1) of each muscle within each synergy, where 0 is no contribution and higher values indicate a higher contribution. These weights were different between stroke subjects and controls for the contralesional (DOT_GROUP_ pathology effect: F(2,47) = 42.21, *p* < 0.001) and ipsilesional arm (pathology effect: F(2,47) = 35,41, *p* = 0.001); see Figure 6A. Specifically, for stroke subjects, the DELT-ant had a higher contribution (weight) in the first synergy, the DEL-pos had a higher contribution in the third synergy, while RHOM and INFR had a lower contribution in the first synergy and higher in the third synergy in both arms. Furthermore, the LAT had a higher contribution in the first synergy only for the AF and RF tasks. There were no main effects due to the side of the lesion (F(1,23) = 1.28, *p* = 0.651; Figure 6A). However, between LBD and RBD subjects, there was an arm-dependent difference (interaction effect: side of lesion x arm effect–F(1,23) = 31.51, *p* = 0.032) and task-dependent difference (interaction effect: side of lesion x task–F(2,47) = 25.46, *p* = 0.010). As for the former, their synergy organization was more similar in the ipsilesional arm than in the contralesional arm. As for the latter, their synergy organization was more similar in the presence of either assistive or resistive forces than in the absence of force (interaction effect: side of lesion x task–F(2,47) = 25.46, *p* = 0.010).

In general, in the absence of force fields, when comparing the LBD and RBD groups, the weight coefficients of muscle synergies were less altered for RBD subjects, both for the ipsilesional arm (post-hoc LBD vs. RBD *p* = 0.03) and the contralesional arm (post-hoc LBD vs. RBS *p* = 0.04). Instead, when forces were present, the difference tended to disappear in the ipsilesional arm (post-hoc AF: *p* = 0.436 and RF: *p* = 0.101) and to be inverted in the contralesional arm: the weight coefficients were more altered for the RBD than the LBD group (post-hoc AF: *p* = 0.04 and RF: *p* = 0.02); see Figure 6A. The similarity of the weight coefficients between the two arms was lower in the stroke subjects with respect to controls in all tasks (DOT_ARM_-pathology effect: F(2,47) = 4.64, *p* = 0.021; Figure 6B) and differed also between RBD and LBD subjects (post-hoc LBD vs. RBD: *p* = 0.041), i.e., RBD subjects had a more similar structure of the muscle synergies than LBD (Figure 6B). This difference was evident in the BB-long and BB-short in the fourth synergy and in the TRAP in the fifth synergy, particularly in AF and NF tasks (Supplementary Material, Figures S3 and S4).

#### 3.3.2. Activation Coefficients of Muscle Synergies

In stroke subjects and controls, the activation coefficient of each synergy was modulated across directions, so that its engagement was specific to one or two consecutive reaching directions and the activations of the whole set of muscle synergies allowed coverage of the entire workspace (Figure 5B), in particular:

- Synergy S1 was mainly active during movements directed toward 0° and 315°;
- Synergy S2 was mainly active during movements directed toward 90° and 135°;
- Synergy S3 was mainly active during movements directed toward 45° and 90°;
- Synergy S4 was mainly active during movements directed toward 180° and 225°;
- Synergy S5 was mainly active during movements directed toward 225°, 270°.

The activation coefficients (H) described the temporal activation profile of each synergy and were altered for both arms in stroke survivors with respect to the controls in all tasks, as highlighted by the Pearson correlation coefficient (r_GROUP_-F(2,47) = 46.16, *p* < 0.001 for the contralesional and F(2,47) = 35.62, *p* = 0.001 for the ipsilesional arm; Figure 6C). There was not a main effect of the side of the lesion (side of lesion effect: F(1,23) = 4.31, *p* = 0.510, Figure 6C). In general, stroke subjects had lower values for the activations of the first synergy H1 during movements directed toward 0°/315°, and for the activations of the fourth synergy H4 during movements directed toward 180°/225° (Figure 5B). As expected, the difference between the two arms of each subject in terms of activation coefficients was greater in stroke subjects than in controls for all tasks (r_ARM_-pathology effect: F(2,47) = 50.24, *p* = 0.012; see Figure 6D).

Moreover, for both arms of stroke subjects, the similarity of the activation coefficients with those of the controls was task-dependent (r_GROUP_-ipsilesional arm task effect: F(2,47) = 11.85, *p* = 0.023; contralesional arm task effect: F(2,47) = 14.54, *p* = 0.02; Figure 6C), as well as the similarity of the activation coefficients of the two arms for all populations (r_ARM_-task effect: F(2,47) = 11.74, *p* = 0.021, Figure 6D). Specifically, this similarity was slightly higher when interacting with the resistive force field than in the other tasks.

## 4. Discussion

This study systematically investigated the differences in movement execution, spinal maps and muscle synergies in a population of twenty-five chronic hemiparetic stroke subjects and their age- and sex-matched controls during point-to-point planar reaching movements. We used a robotic manipulandum as a haptic device to provide three different mechanical environments and as an instrument to quantify movement performance jointly with the measurement of non-invasive upper limb EMG activity. We included stroke subjects with left and right hemiparesis with the goal of characterizing their motor and muscular alterations in both the ipsilesional and contralesional upper limb. We described how upper limb motor performance, spinal maps and muscle synergies were different from those of controls in both upper limbs, although less altered in the ipsilesional than in the contralesional arm. We observed that left-hemiparetic stroke subjects (RBD) had worse movement execution performance in both arms with respect to LBD and controls. However, in the absence of forces, their muscle synergies’ organization in the ipsilesional side, which, for them, was the right-dominant, was more preserved with respect to the LBD subjects, who underwent a deeper reorganization since they were forced to use the left, non-dominant arm more in everyday life tasks.

### 4.1. Stroke Subjects Had Different Performance than Controls and the Differences Were Dependent on the Side of the Brain Lesion

The movements of chronic stroke subjects were slower, less smooth and less accurate with respect to those of controls, in accordance with previous studies [24,38,41,42,83,84,85]. The movement performance was altered also in the ipsilesional side, although to a lesser extent than in the contralesional side [83,86,87,88].

As for the influence of the brain lesion, in our study, the LBD subjects had more difficulty in movement planning, i.e., feedforward control, while the RBD subjects had difficulty in movement execution. The model of brain lateralization developed by the Sainburg group [38,83] predicts that a lesion in the left hemisphere determine deficits in specifying initial trajectory features, while a lesion in the right hemisphere results in final position accuracy [89]. If the abovementioned predictions were valid for the ipsilesional arm of stroke survivors, this would support not only the hypothesis that there is a lateralized brain specialization [38,39,40,89], but also the idea that the ipsilesional hemisphere contributes to arm movements. Schaefer et al. [41] found evidence supporting these predictions and our results speak in favor of their hypotheses, although our tasks were different and parts of the related performance indexes were not directly comparable. Specifically, in our case, the lateral deviation and smoothness could account for difficulty in the final phase of movement execution, but we had no direct measure of final error since subjects were required to stay inside the target at the end of each trial.

Finally, an important element of novelty of the present study is the investigation of the same reaching task in the presence of different force fields. As expected, the assistive force field helped all subjects to improve their performance. In general, the differences in motor performance between stroke survivors and controls and between RBD and LBD groups as well as between RBD and LBD groups were more evident in free space than in the presence of either an assistive or a resistive force.

### 4.2. Stroke Altered Spinal Maps for both the Contralesional and Ipsilesional Arm

The estimated MN activity represents a useful tool to explore muscle organization when the task is complex and involves the activity of several muscles. Few studies [37,52] applied this method to upper limb movements of control and stroke subjects. Our results show that spinal maps were sensitive to stroke both for the ipsilesional and contralesional upper limbs. However, they did not detect differences between RBD and LBD subjects, i.e., they were not sensitive to the effect of the site of stroke lesion during upper limb movements.

### 4.3. Upper Limb Muscle Synergies both in the Ipsilesional and Contralesional Side Were Significantly Altered with Respect to Controls in Terms of their Weight and Activation Coefficients, but Not in Terms of Dimensionality. The Structure of the Muscle Synergies Had Alterations Dependent on the Brain Hemisphere Side of the Lesion

We found no significant difference in the number of muscle synergies between controls and stroke subjects both in the ipsilesional and contralesional side. This result partially disagrees with the existing literature [18,90]. In severe chronic post-stroke individuals, the number of muscle synergies is correlated with spasticity [90], reduced walking speed, clinical measures of balance and walking function, biomechanical measures such as propulsion and step length asymmetry [54,56], and with the outcome of the Fugl-Meyer assessment [18]. However, the number of muscle synergies might not change in a task with compensation of gravity that allows stroke subjects with more severe impairment to perform planar reaching movements that might be impossible otherwise. Indeed, Tropea et al. [23] adopted a very similar task to ours, where the arm was supported by a planar robotic device, and they also did not find a different number of muscle synergies between controls and the stroke population. Our results expand this evidence, showing that the arm support against gravity might favor the preservation of the number of muscle synergies also between the ipsilesional and contralesional arm.

For both populations, three synergies primarily involved shoulder muscles, one synergy involved distal muscles and one the trunk muscles, as also observed by other authors [78,91]. We found a different organization as described by the weight coefficients between controls and stroke subjects and between sides in the stroke population. Differently from Cheung et al. [71], we found that the weight coefficients of muscle synergies are also altered in the ipsilesional side with respect to controls. This difference might be due to the level of impairment: indeed, in our population, there were several subjects with more severe impairment than in Cheung et al. [71].

Evidence of impairment of muscle synergies in the ipsilesional side has never been reported before in the stroke population because all the other existing works focused mainly or only on upper limb muscle synergies in the contralesional side [18,19,23,90].

Concerning the contralesional side of post-stroke subjects, our results are in agreement with the findings of Tropea et al. [23], who also found a different organization of muscle synergies after stroke, especially the ones related to the shoulder muscles. While the alteration of muscle synergies in the contralesional is in agreement with the findings of Tropea et al. [23], their alterations also in the ipsilesional arm have never been clearly reported. Moreover, while [23] adopted a task equivalent to our NF task, we observed that the similarity of muscle synergies between controls and the stroke population depends on the task: when the reaching movements are in the presence of assistive or resistive forces, the muscle synergies become more similar, both in terms of structure and activations.

Finally, when moving in the absence of force fields, the LBD group had weight coefficients of muscle synergies more altered than the RBD group in both arms. The presence of force fields reduced and tended to invert this behavior, respectively, in the ipsilesional and in the contralesional arm. The LBD group had also weight coefficients more similar between body sides in all tasks than the RBD group. This suggests that brain reorganization and compensatory strategies can induce a relevant bilateral reorganization of muscle coordination that is not mirrored with respect to the side of lesion but is influenced by handedness and/or hemispheric specialization.

Cheung et al. did not find significant evidence [18] of an influence of the hand dominance and lesion side on the similarity in the number of muscle synergies between contralesional and ipsilesional arms, as well as on the similarity between the muscle synergies in the contralesional arm. Our results suggest that the effect of the lesion side on upper limb weight coefficients of muscle synergies after stroke might emerge when movements are performed without the need of compensating for gravity, allowing also more impaired subjects to perform the tasks, and in the absence of relevant external forces.

### 4.4. Implication for Research Studies, Assesment and Rehabilitation of Chronic Subjects

The ipsilesional upper limb of chronic stroke subjects is not “unaffected”, as commonly thought, but it presents alterations in terms of motor performance, muscle activity and synergies. This implies that studies focused on contralesional upper limb performance and muscle coordination after stroke should be based on the comparison with the corresponding arm of unpaired controls, or, when comparing the two arms of the same stroke subjects, we should take into account to what extent the ipsilesional side is also affected by stroke. Additionally, the lesion side should be carefully considered since it might influence the above-mentioned measures.

In this framework, the analysis of kinematics, spinal maps and muscle synergies with the metrics proposed here has the potential to be a useful quantitative framework for the investigation and the quantitative assessment of upper limb deficits after stroke. They allowed identification of specific biomarkers of stroke but also differences between subjects with left and right brain lesions (with the exception of the spinal maps for the latter).

In summary, this study might work as a pilot study to instruct other researchers that use similar robotic devices not only to measure performance scores but also muscle synergies and spinal activity. Such assessment might be an opportunity for detecting underlying impairments that might not be evident when measuring performance.

As for the rehabilitation implications, such a framework, providing integrated measures from different sources, has the potential to increase the understanding of recovery mechanisms at different levels, facilitating the development of tailored interventions. In fact, for planning rehabilitative intervention, the heterogeneity of the stroke population is a problem [92]. Previous studies have already demonstrated a link between kinematic assessment and clinical scales [93,94] and the need to account also for different deficits induced by right or left hemisphere damage [90]. However, in neurorehabilitation, focusing only on the performance measures to modulate the rehabilitative task, as most technological approaches do, overlooks the importance of controlling the development of motor and muscle strategies, if possible, facilitating some and inhibiting others. Having measures of muscle activation in terms of synergies and spinal activity after a stroke will help in exploiting the motor system redundancy to facilitate customization and the target of individual goals and harnessing neuroplasticity, as illustrated in [92].

Finally, modifying the mechanical environments of the task can change muscle synergy structures and activations in stroke survivors and controls, since the first exposure to the forces. However, it is not yet clear which are the rehabilitative goals in terms of muscle coordination—for example, whether to promote muscle synergies resembling the ones of healthy individuals or new ones [95,96]. Preliminary evidence in acute stroke patients suggests that during motor recovery, altered muscle synergies tend to resemble the ones of healthy individuals [23,97]. This could be taken into account, further explored and exploited in robot-based rehabilitation protocols to maximize functional recovery after neurological injuries and better enable rehabilitation professionals to devise treatment plans based on the residual individual abilities.

### 4.5. Limitations of the Study

Stroke subjects are an extremely heterogeneous population; therefore, a larger number of subjects [98] with more diverse motor impairments, lesion sizes and sites, etc., is necessary to confirm the results of this study. The RBD group had a higher degree of spasticity than the LBD group in the contralesional side, as measured with the MAS for the muscles controlling the shoulder joint motion, and this could affect the results related to behavioral performance and muscle activations for this side of the body. Instead, since the task was unimanual, we expected a lower impact of this difference on our main results related to the ipsilesional arm. In the following, we discuss in detail each of our main results in relation to this difference.

Although we acknowledge that spasticity on the contralesional arm might influence motor performance also in the ipsilesional arm, our main result that in the ipsilesional arm, the LBD population had higher aiming errors, while RBD had higher lateral (execution) errors during reaching movements, is in line with the prediction of the lateralization model proposed by Sainburg and colleagues [38,40]. More specifically, our result confirms the findings obtained by this group [39,84] and it seems rather to support the hypothesis of hemispheric specialization in movement control [40,99] than being a mere byproduct of the difference in spasticity between the LBD and RBD groups. As for the spinal maps, since we did not detect differences between RBD and LBD subjects, their difference in spasticity did not affect this result. Instead, the alterations at the level of the muscle synergies in both arms could be either due to or be a combination of i) the brain lateralization that determined also the performance difference between the two groups and ii) the difference in contralesional spasticity measured at the shoulder. In conclusion, spasticity is an important factor to be considered when interpreting our results and should be appropriately measured and carefully taken into account when planning similar future experiments.

We also acknowledge that we adopted a self-selected speed rather than a fixed speed to favor the execution of natural reaching movements. Reaching speed has an impact on upper limb muscle synergies [78,100]; therefore, we cannot exclude the possibility that this approach might have influenced our results. However, forcing the controls or stroke subjects to execute the planar reaching movements at a fixed speed would have altered the execution of movements in a natural way. The arm support provided by the robotic manipulandum has also an influence on upper limb muscle synergies [79].

Therefore, our analysis could be extended to upper limb 3D reaching movements, with gravity effect, at different controlled speeds, sides and different environments (such as variable assistance and constant resistance), to confirm and complete our results.

## Figures and Tables

**Table 1:** Clinical data of the stroke population.

		S	PH	E	AGE (ys)	DD (ys)	Site of Lesion	FMA-UE	MAS	
								A–D (0–66)	Shoulder	Elbow
RBD	S01	F	L	H	65	16	Right occipital	42	1	1
	S02	F	L	H	64	10	Right fronto-parietal pre-rolandic	17	2	1
	S03	M	L	H	67	13	Right fronto-parietal	5	3	3
	S04	M	L	I	57	1	Right basal ganglia, temporal lobe and insula	6	1.5	1.5
	S05	F	L	I	40	11	Right complete middle and anterior cerebral arteries	9	1.5	2
	S06	M	L	H	61	8	Right fronto-parietal	39	1.5	1
	S07	M	L	I	67	1	n.a.	26	1.5	1.5
	S08	M	L	H	68	1.5	Right basal ganglia	40	3	2
	S09	F	L	H	57	1.9	Medial right frontal lobe, cingulate cortex and corpus callosum	57	2	0
	S10	M	L	I	48	1	Right fronto-temporal and insula	19	3	3
	S11	M	L	H	50	2	Right frontal, temporal lobes and basal ganglia	26	2	1
	S12	F	L	H	74	3.5	Right basal ganglia and posterior internal capsule	32	2	2
	S13	M	L	H	69	2	Right frontal and insula lobes	8	2	3
	S14	M	L	I	63	11	Right caudal and lateral part of the pons	58	2	1.5
LBD	S15	F	R	I	40	10	Left basal ganglia, internal capsule and parietal lobe	22	0	1
	S16	M	R	H	62	3	Left basal ganglia	14	2	2
	S17	F	R	I	61	7	Left basal ganglia, internal capsule, temporal lobe and insula	21	1	3
	S18	F	R	I	44	12	n.a.	18	1	1.5
	S19	M	R	I	68	2	Left internal capsule and basal ganglia	25	1.5	2
	S20	M	R	H	67	27	Left frontal lobe	5	2	3
	S21	M	R	I	55	2	Left frontal and parietal lobes, insula, cerebral peduncle, internal capsule	20	0	0
	S22	M	R	I	78	8	Left cerebellum, midbrain, occipital lobe, cerebral peduncle	33	1.5	1.5
	S23	F	R	I	68	12	Left basal ganglia, internal capsule and occipital lobe	57	1	0
	S24	M	R	I	59	2	Left lenticular nucleus and corona radiata	63	0	0
	S25	M	R	I	59	0.6	Left frontal, parietal, temporal and insular lobes and basal ganglia	16	2	3
		9F/16M	11R/ 14L	14I/ 11H	60.4 ±10	6.7 ±6.4		27.1 ±17.5	1.6 ±0.8	1.6 ±1

RBD = Right brain damage; LBD = Left brain damage; S = Sex: Female/Male; PH = Paretic (i.e., contralesional) hand: Right (R)/Left (L); E = Etiology: Ischemic (I)/Hemorrhagic (H); DD = disease duration (years); FMA-UE = Fugl-Meyer Assessment for upper extremity; MAS = Modified Ashworth Scale: 0 = normal function; 4 = severe spasticity. n.a. = not available

**Table 2:** Kendall’s chart for the computation of spinal maps [73].

	DELT-Ant	PECT	LAT	INFR	RHOM	BB-Short	BB-Long	PRON	BRAD	TB-Lat	TB-Long	DELT-Mid	DELT-Post	TRAP	FLEX	EXTE
C2														x		
C3														X		
C4				x	x									X		
C5	X	X		X	X	X	X		X			X	X			x
C6	X	X	X	X		X	X	X	X	x	x	X	X		X	X
C7		X	X					X		X	X				X	X
C8		X	X							X	X				x	x
T1		X								x	x					

X corresponds to a weight coefficient of 1 and x corresponds to a weight coefficient of 0.5. DELT-an = anterior deltoid; PECT = pectoralis major; LAT = latissimus dorsi; INFR = infraspinatus; RHOM = rhomboid major; BB-short = Biceps Brachii short head; BB-long = Biceps Brachii long head; PRON = pronator teres; BRAD = brachioradialis; TB-lat = Triceps Brachii lateral head; TB-long = Triceps Brachii long head; DELT-mid = medial deltoid; DELT-post = posterior deltoid; TRAP = upper trapezius; FLEX = flexor carpi radialis; EXTE = extensor carpi radialis.

## Data Availability

The data presented in this study are available on request from the corresponding author. The data are not publicly available due to patient privacy considerations (HIPPA).

## Supplementary Materials

The following are available online at https://www.mdpi.com/article/10.3390/life11050383/s1, Figure S1: The spinal maps obtained for the eight targets in the null force (NF, panel A) and assistive force (AF, panel B) tasks, Figure S2: Number of synergies, Figure S3: Weight and activation coefficients of muscle synergies during the assistive task (AF), Figure S4: Weight and activation coefficients of muscle synergies during the null force task (NF). Table S1: rANOVA ipsilesional arm, Table S2: rANOVA contralesional arm, Table S3: rANOVA LBD and RBD subjects, Table S4: rANOVA on arm similarity parameters.
