# Supplementary material for: Effects of Hemispheric Stroke Localization on the Reorganization of Arm Movements within Different Mechanical Environments
Source: Life (Basel). 2021 Apr 23;11(5):383. doi: 10.3390/life11050383 (PMC8145329; doi:10.3390/life11050383)
Supplement: Supplementary file 1 [file life-11-00383-s001.zip › life-1166357-supplementary.pdf]

Effects of Hemispheric Stroke Localization on the Reorganization of Arm Movements within Different Mechanical Environments

Laura Pellegrino [1,+], Martina Coscia [2,3,+], Camilla Pierella [1,4,*], Psiche Giannoni [1], Amel Cherif [1,5], Maddalena Mugnosso [1,5], Lucio Marinelli [4,6] and Maura Casadio [1,*]

1 Department Informatics, Bioengineering, Robotics and Systems Engineering (DIBRIS), University of Genoa, 16126 Genoa, Italy; laura.pellegrino@edu.unige.it (L.P.); psiche.giannoni@gmail.com (P.G.); amel.cherif@iit.it (A.C.); maddalena.mugnosso@iit.it (M.M.)
2 Bertarelli Foundation Chair in Translational Neuroengineering, Ecole Polytechnique Federale de Lausanne, 1015 Lausanne, Switzerland; martina.coscia@virgilio.it
3 Wyss Center for Bio- and Neuroengineering, 1202 Geneva, Switzerland
4 Department of Neurosciences, Rehabilitation, Ophthalmology, Genetics, and Maternal and Children's Sciences (DINOGMI), University of Genova, 16126 Genoa, Italy; lucio.marinelli@unige.it
5 Robotics, Brain and Cognitive Sciences Department, Istituto Italiano di Tecnologia, 30 16163 Genoa, Italy
6 Division of Clinical Neurophysiology, Department of Neuroscience, IRCCS Ospedale Policlinico San Martino, 16132 Genoa, Italy
* Correspondence: camilla.pierella@edu.unige.it (C.P.); maura.casadio@unige.it (M.C.); Tel.: +390103532749
† These authors contributed equally

Mauchly-test statistics of sphericity assumption and Greenhouse–Geisser correction

Table S1: rANOVA ipsilesional arm.

Indicators	Effect	χ^2	df	p-value	ε
Average Speed	Task x pathology	27.67	2	0.001	0.68
100 ms-Aiming error	Task x pathology	18.50	2	0.009	0.72

Table S2: rANOVA contralesional arm.

Indicators	Effect	χ^2	df	p-value	ε
Average Speed	Task x pathology	20.59	2	0.002	0.73
Smoothness index	Task x pathology	36.55	2	0.001	0.64
Lateral deviation	Task x pathology	18.67	2	0.008	0.73

Table S3: rANOVA LBD and RBD subjects.

Indicators	Effect	χ^2	df	p-value	ε
Smoothness index	Arm x side of lesion	18.09	2	0.001	0.60
Lateral deviation	Task x side of lesion	11.80	2	0.002	0.70
DOT_{GROUP}	Arm x side of lesion	46.33	2	0.023	0.72

Table S4: rANOVA on arm similarity parameters.

Indicators	Effect	χ^2	df	p-value	ε
Q_{2D-ARM}	Task	21.54	2	0.021	0.65
r_{ARM}	Task	3.45	2	0.001	0.86

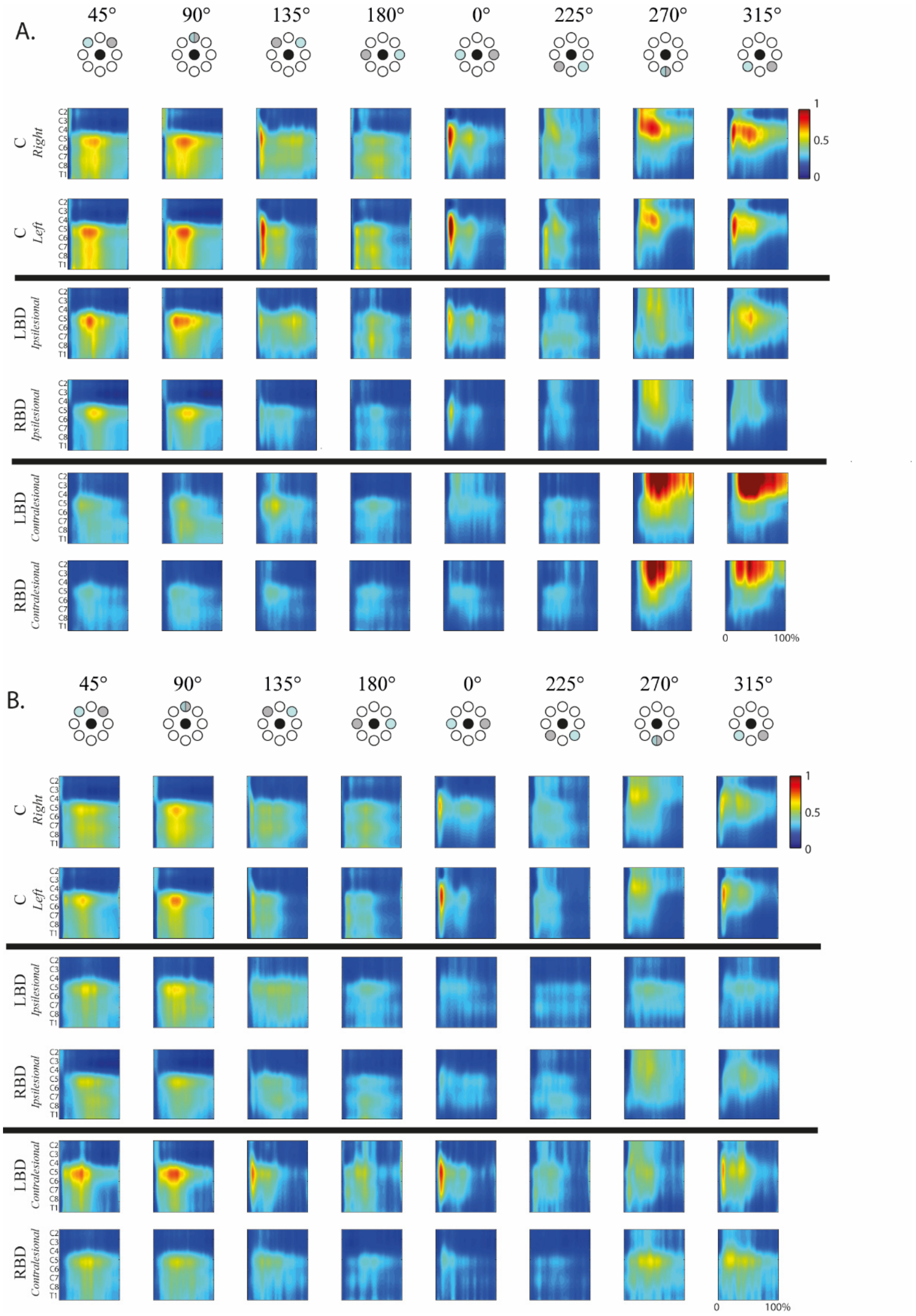

Figure S1: The spinal maps obtained for the eight targets in the null force (NF, panel A) and assistive force (AF, panel B) tasks. The first two rows are referred to the right and left arm of control subjects (C), the third and the forth rows to the ipsilesional (non-paretic) arms of stroke subjects with respectively left brain (LBD) and right brain (RBD) damage, while the fifth and sixth rows to their corresponding contralesional (paretic) arms. On the x-axis the movement duration is represented in percentage. Spinal maps are referred to equal movements in the joint space, i.e. for each column the top panel indicates the corresponding target directions (grey target) for the right arm, while the corresponding target directions of the left arm were mirror symmetric with respect to the vertical midline.

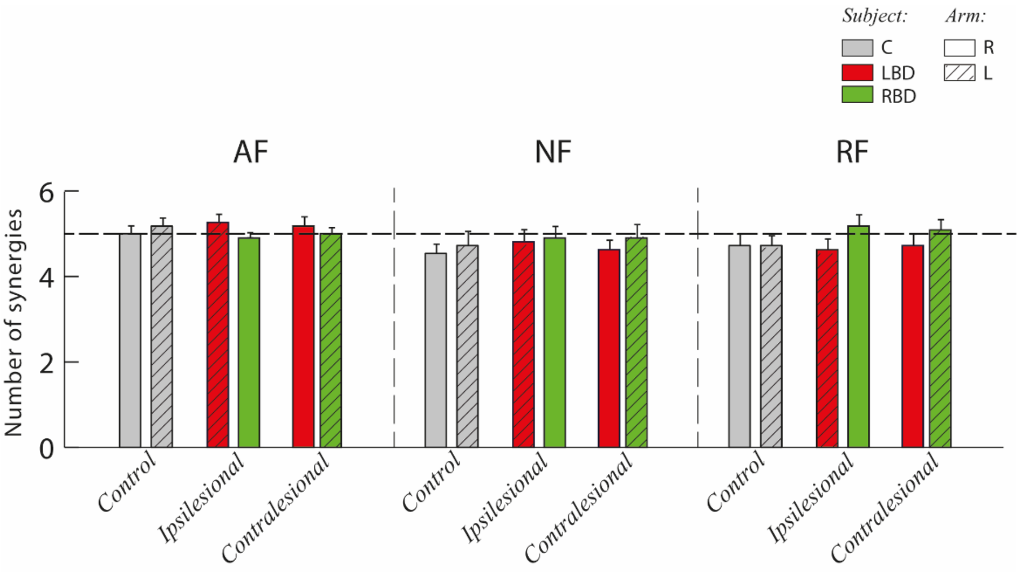

Figure S2. Number of synergies for controls (C, grey bars) and stroke subjects with right brain damage (RBD, green bars), and left brain damage (LBD, red bars) during motion in presence of assistive (AF), resistive (RF) and in absence of external force (NF) in the right (bars with uniform color) and left (bars with diagonal lines) arm. The error bars indicate the standard error. The contralesional arm (paretic) corresponds to the left (L) arm in RBD subjects and to the right (R) arm in LBD subjects. The ipsilesional arm (non-paretic) corresponds to the right (R) arm in RBD subjects and the left (L) arm in LBD subjects.

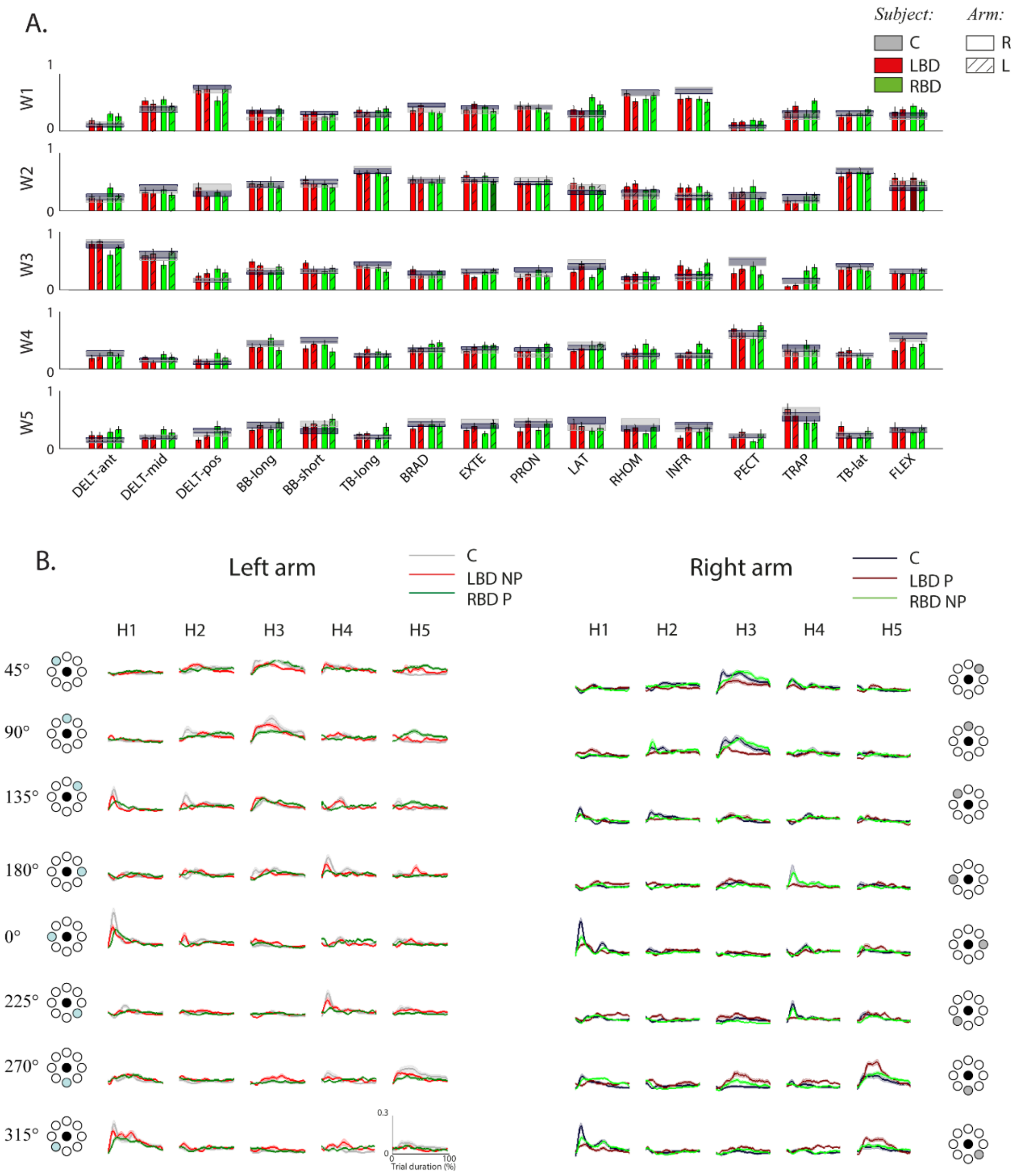

Figure S3. Weight and activation coefficients of muscle synergies during the assistive task (AF). Panel A: Weight coefficients for all muscle synergies (W1 to W5) for the two arms (right: bars with uniform color; left: bars with diagonal lines). Controls (C) and stroke subjects with right brain damage (RBD), and left brain damage (LBD) are shown with different colors as indicated in the legend. The error bars represent the standard error. Panel B: Mean activation profiles for C (grey), RBD (green), and LBD (red) for all muscle synergies (H1 to H5) in the right (right panel) and left (left panel) arm. The shaded area indicates the standard error. Right and left activation profile coefficients are referred to equal movements in the joint space; i.e. for each column the left panel indicates the corresponding target directions (grey target) for the right arm, while the corresponding target directions of the left arm were mirror symmetric with respect to the vertical midline.

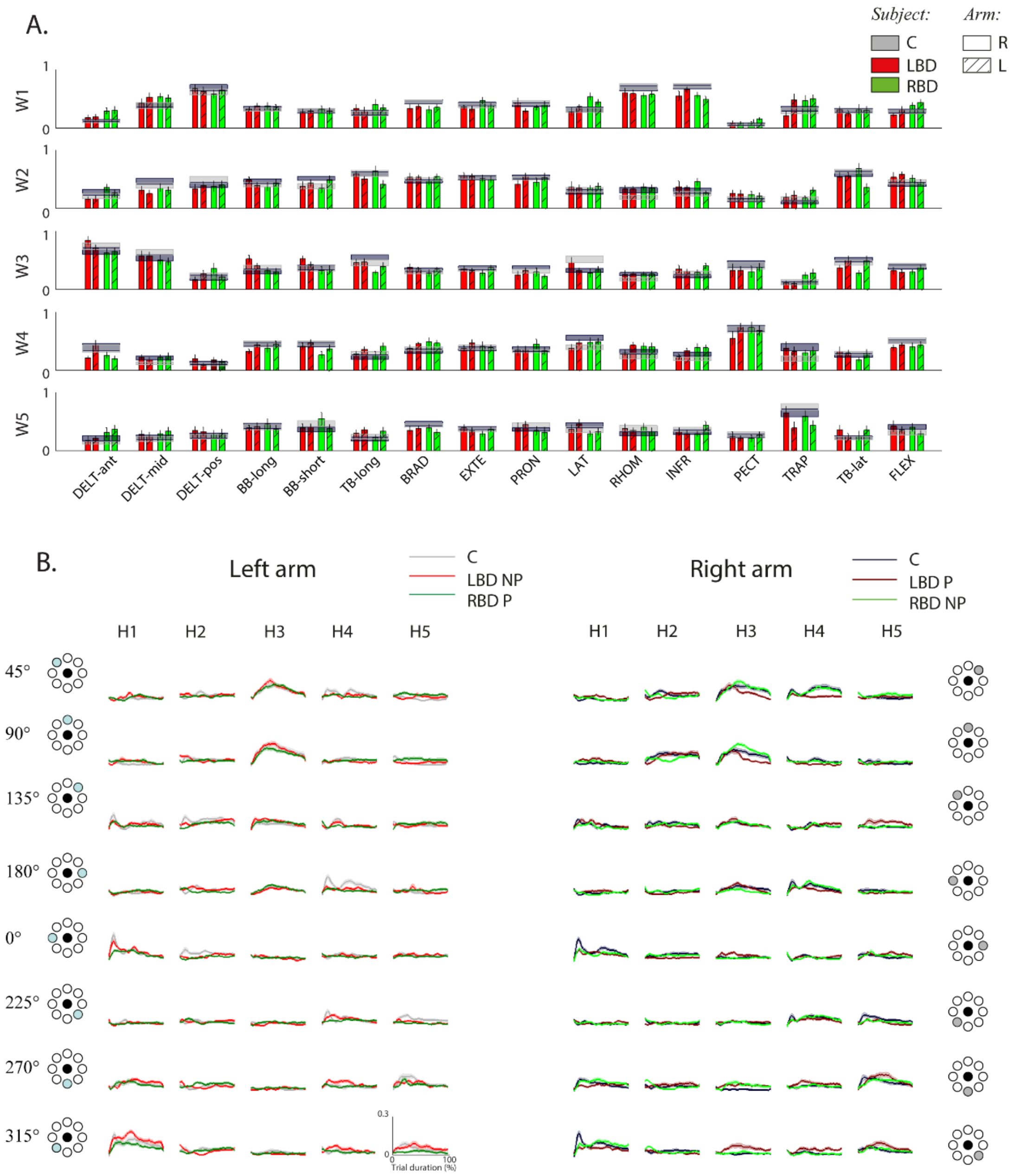

Figure S4. Weight and activation coefficients of muscle synergies during the null force task (NF). Panel A: Weight coefficients for all muscle synergies (W1 to W5) for the two arms (right: bars with uniform color; left: bars with diagonal lines). Controls (C) and stroke subjects with right brain damage (RBD), and left brain damage (LBD) are shown with different colors as indicated in the legend. The error bars represent the standard error. Panel B: Mean activation profiles for C (grey), RBD (green), and LBD (red) for all muscle synergies (H1 to H5) in the right (right panel) and left (left panel) arm. The shaded area indicates the standard error. Right and left activation profile coefficients are referred to equal movements in the joint space, i.e. for each column the left panel indicates the corresponding target directions (grey target) for the right arm, while the corresponding target directions of the left arm were mirror symmetric with respect to the vertical midline.
